# Elevated pulsatility index is associated with poor functional outcome in stroke patients treated with thrombectomy: A retrospective cohort study

**DOI:** 10.1111/cns.13888

**Published:** 2022-06-16

**Authors:** Wenbo Zhao, Ran Liu, Wantong Yu, Longfei Wu, Chuanjie Wu, Chuanhui Li, Sijie Li, Jian Chen, Haiqing Song, Yang Hua, Qingfeng Ma, Xunming Ji

**Affiliations:** ^1^ Department of Neurology, Xuanwu Hospital, Beijing Institute for Brain Disorders Capital Medical University Beijing China; ^2^ Beijing Key Laboratory of Hypoxic Conditioning Translational Medicine, Xuanwu Hospital Capital Medical University Beijing China; ^3^ Department of Vascular Ultrasound, Xuanwu Hospital Capital Medical University Beijing China; ^4^ Department of Neurosurgery, Xuanwu Hospital Capital Medical University Beijing China

**Keywords:** “no‐reflow” phenomenon, acute ischemic stroke, endovascular thrombectomy, microcirculation no‐reperfusion, pulsatility index

## Abstract

**Aims:**

To evaluate pulsatility index (PI) in patients with acute ischemic stroke (AIS) who underwent endovascular thrombectomy (EVT).

**Methods:**

Patients were retrospectively recruited if their stroke were secondary to middle cerebral artery (MCA) occlusion and achieved full recanalization after EVT. Transcranial Doppler was performed within 24‐hour post‐EVT. The primary outcome was correlation between the MCA‐PI on the affected side and 3‐month functional outcome, with modified Rankin scale (mRS) ≥5 indicated extremely poor functional outcomes.

**Results:**

Totally, 170 patients were included. High MCA‐PI was positively related to the 3‐month mRS score (*r* = 0.288, *p* < 0.001). The highest quartile of the MCA‐PI was associated with a high incidence of extremely poor functional outcomes (adjusted OR, 13.33; 95% CI, 2.65–67.17; adjusted *p* = 0.002) after adjusting for confounding factors. The predictive capacity of the MCA‐PI for extremely poor functional outcomes was good (area under the curve, 0.755; 95% CI, 0.655–0.854; *p* < 0.001), and its cutoff value for predicting extremely poor outcomes was 1.04, with a sensitivity of 65.6% and specificity of 78.3%.

**Conclusion:**

The MCA‐PI on the affected side is a prognostic biomarker in patients who have undergone stroke thrombectomy. An elevated MCA‐PI may be prognostically valuable for predicting extremely poor functional outcomes.

## Graphical abstract

The affected middle cerebral artery pulsatility index was significantly correlated with 90‐day functional outcomes assessed by modified Rankin scale. Receiver operating characteristic curve for extremely poor functional outcomes 3 months after thrombectomy demonstrating area under cure was 0.755 with *p* < 0.001. The optimal cutoff point for predicting poor outcome was 1.04, with sensitivity of 0.656 and specificity of 0.783.

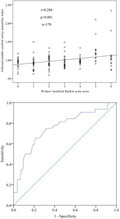

## 1 INTRODUCTION

Endovascular thrombectomy (EVT) has been demonstrated to be an effective therapy for patients with acute ischemic stroke (AIS) caused by proximal large vessel occlusions. It can yield recanalization rates of >80% as compared to traditional therapies.^1^, ^2^, ^3^ However, previous imaging studies have found that microcirculation no‐reperfusion after recanalization of proximal large vessels, namely the “no‐reflow” phenomenon, is a common.^4^, ^5^ Its incidence ranges from 25% to 81% depending on the individual and the evaluation methods used, and this phenomenon has been demonstrated to be associated with unfavorable outcomes.^6^, ^7^ Currently, perfusion imaging methods, including CT perfusion imaging, perfusion‐weighted imaging, and single‐photon emission CT, are often used to evaluate the reperfusion status after reperfusion therapy.^6^, ^8^ However, these examinations are difficult to perform timely and repeatedly after EVT, which may render the prevention or counteraction of post‐EVT no‐reflow impossible.^9^

Transcranial Doppler (TCD) ultrasonography is an inexpensive and noninvasive technology that allows repeated investigations of rapid changes in cerebral perfusion in clinical practice.^10^ The Gosling pulsatility index (PI) derived from TCD has been recognized as a measure of distal cerebrovascular resistance in ischemic stroke^11^, ^12^, ^13^ and a clinical marker of the no‐reflow phenomenon after reperfusion therapy.^14^ Moreover, the PI in patients with AIS treated with intravenous thrombolysis was found to be associated with clinical outcomes.^15^ However, it remains unclear whether the PI is associated with the functional outcomes of patients with AIS treated with EVT.

In this study, we aimed to investigate the association of the middle cerebral artery (MCA)‐PI on the affected side detected early after EVT with the functional outcomes of patients with AIS caused by MCA occlusion in whom excellent recanalization (thrombolysis in cerebral infarction [TICI] grade 3) was achieved. If such an association existed, we further sought to determine the cutoff value of the MCA‐PI for predicting the functional outcomes.

## 2 METHODS

### 2.1 Study design and participants

This cohort study was based on a prospective registry study conducted at the Xuanwu Stroke Center, a high‐volume stroke center in northern China. The details of the registry study and the intervention processes of EVT for AIS at our institution have been described previously.^16^, ^17^ All patients with AIS treated with EVT between January 2013 and December 2020 were screened. The protocol of this study was approved by the Ethics Committee of Xuanwu Hospital, Capital Medical University. All patients or their legally authorized representatives provided written informed consent upon admission to the hospital.

The inclusion criteria for this study were as follows: (1) aged≥18 years at the stroke onset; (2) AIS caused by MCA occlusion in the M1 segment and treated with EVT; and (3) the culprit vessel was completely recanalized (TICI grade (3) as confirmed on the last digital subtraction angiography image. Meanwhile, the exclusion criteria were as follows: (1) concomitant ipsilateral internal carotid artery or MCA stenosis, (2) TCD after EVT was not performed, (3) poor or no acoustic windows that challenge the TCD results, and (4) reoccluded culprit MCA detected on post‐EVT TCD, CT angiography, or magnetic resonance angiography.

### 2.2 TCD ultrasonography

According to the local vascular ultrasound department regulations, all patients with AIS treated with EVT underwent TCD ultrasonography performed by experienced sonographers within 24 h after EVT. TCD was performed using 1.6‐MHz pulsed‐wave Doppler probes, while the transcranial spectral signals derived from the systolic, diastolic, and mean flow velocities (cm/s) were acquired and analyzed using a multichannel Doppler unit (Delica EMS‐9 PB; Shenzhen Delica Medical Equipment Co., Ltd.). The PI was calculated by subtracting the end‐diastolic velocity from the peak systolic velocity and then dividing the difference by the mean flow velocity. Bilateral MCA signals were acquired through the temporal acoustic bone window at a depth of 50–60 mm.

### 2.3 Data collection

Baseline data on demographic characteristics, medical history, lifestyle risk factors, and drugs used before stroke were collected at hospital admission using a standard questionnaire. Stroke neurologists assessed each patient's stroke severity at admission using the National Institutes of Health Stroke Scale (NIHSS). The Alberta Stroke Program Early CT Score (ASPECTS) was evaluated on initial CT at admission by radiologists and stroke neurologists, and all other imaging results after EVT were evaluated by radiologists. Clinical laboratory measurements, including the levels of serum lipids, plasma glucose, and biomarkers of the liver and kidney, were obtained at admission. The EVT operational details and related time points were documented by the interventionists. The patients were recommended to visit the hospital for follow‐up assessment of the functional outcomes at 3 months, while those unable to return to the hospital were interviewed via telephone by trained neurologists using a structural questionnaire.

All the aforementioned variables were documented in the database and collected for this study. Initially, the parameters of TCD ultrasonography were not included in the database. These parameters were retrospectively collected with the unique identifier of each participant and added to the database.

### 2.4 Outcome assessment

The primary outcome of this study was the correlation between the MCA‐PI on the affected side and functional outcome at 3 months after EVT; and functional outcome was assessed by modified Rankin scale (mRS) score. The secondary outcomes were as follows: (1) the association of higher MCA‐PI and functional outcome, and mRS scores of 6 were defined as mortality; mRS scores of 5–6 were defined as extremely poor functional outcome; mRS scores of 4–6 were defined as poor functional outcomes; (2) the association of higher MCA‐PI and intracerebral hemorrhage (ICH) and symptomatic ICH; symptomatic ICH was assessed according to the definition of the European Cooperative Acute Stroke Study III (any apparently extravascular blood in the brain or within the cranium that was associated with clinical deterioration, as defined by an increase of 4 points or more in the score on the NIHSS, or that led to death, was identified as the predominant cause of the neurological deterioration)^18^; (3) the optimal cutoff value MCA‐PI for predicting functional outcome; and (4) risk factors of MCA‐PI on the affected side.

### 2.5 Statistical methods

As the PI indicates distal flow resistance in the microcirculation, the higher the PI, the greater the microcirculation resistance. Therefore, all participants were dichotomized into the highest quartile and the lower three quartiles, and the baseline characteristics and clinical outcomes were compared between the groups. Missing imaging values were analyzed through a multiple imputation procedure with a missing‐at‐random assumption using a regression‐switching approach. The imputation procedure was performed using the adjacent characteristics of subjects, a predictive mean‐matching method for continuous variables, and logistic regression models for categorical variables.

Generally, means ± standard deviations or medians (interquartile ranges [IQRs]) were used to summarize the data on continuous variables, and two‐sided Student's *t*‐tests for independent samples or the Mann–Whitney U tests were performed to detect differences between the groups. Meanwhile, frequencies and percentages were used to summarize the data on binary variables, and chi‐square tests were performed to detect intergroup differences.

Associations between the MCA‐PI on the affected side and the 3‐month functional outcome assessed using the mRS score were determined using Spearman correlation analysis. As the MCA‐PI was positively correlated with the mRS score, mRS scores of 6, 5–6, and 4–6 were reported and compared between those in the highest quartile and those in the lower three quartiles. Odds ratios (ORs) and 95% confidence intervals (CIs) for the functional outcomes were calculated and reported. Unadjusted models and models adjusted for age, pre‐stroke mRS score, ASPECTS, NIHSS score, intravenous thrombolysis, time from onset to recanalization, and symptomatic ICH were used. In addition, ICH and symptomatic ICH were also compared between the highest quartile and the lower three quartiles, and the ORs and 95% CIs were calculated. Unadjusted models and models adjusted for age, ASPECTS, NIHSS score, intravenous thrombolysis, time from onset to recanalization, hypertension, diabetes mellitus, and antiplatelet treatment before stroke onset were then used.

A receiver operating characteristic (ROC) curve was configured to establish the cutoff value of the MCA‐PI on the affected side for optimal prediction of extremely poor functional outcomes (mRS score of 5–6), and the Youden index was used to find the cutoff to balance the sensitivity and specificity.

Adjustment for possible predictors was performed using a linear regression analysis of covariance for the MCA‐PI on the affected side; these possible predictors included age, sex, arterial hypertension, diabetes mellitus, hyperlipidemia, smoking, and laboratory parameters at admission (e.g., plasma glucose level, platelet count, fibrinogen level, medication before stroke onset, baseline NIHSS score, treatment with intravenous thrombolysis, symptomatic ICH, and stroke type according to the Trial of ORG 10172 in Acute Stroke Treatment [TOAST] criteria).

All statistical analyses were performed using IBM SPSS Statistics version 24.0 (IBM Corp.), with a two‐tailed probability value of 0.05 or less considered as statistically significant.

## 3 RESULTS

### 3.1 Study population

Between January 2013 and December 2020, 170 participants with AIS caused by MCA occlusion in the M1 segment who underwent EVT fulfilled the study criteria and were then recruited. The characteristics of the recruited participants are summarized in Table 1. The average age at onset was 64.70 ± 12.69 years; 133 (66.5%) were men; and the median MCA‐PI was 0.96 (IQR, 0.87–1.07). Comparisons were performed between the highest quartile and lower three quartiles dichotomized according to the MCA‐PI on the affected side (1.15 [IQR, 1.09–1.28] vs. 0.92 [IQR, 0.84–0.98], *p* < 0.001). The participants in the highest quartile were more likely to be older than those in the lower three quartiles (69.69 ± 12.89 vs. 62.91 ± 12.17, *p* = 0.002). The proportion of cigarette smokers was lower in the patients in the highest quartile than in those in the lower three quartiles (22.2% vs. 41.6%, *p* = 0.021) (Table 1).

**TABLE 1:** Characteristics of participants according to quartiles of the pulsatility index values

Patients, *n*	Total	MCA‐PI		*p* Value
		Highest quartile	Lower 3 quartiles	
	170	45	125	
Affected MCA‐PI	0.96 (0.87–1.07)	1.15 (1.09–1.28)	0.92 (0.84–0.98)	<0.001
Age, years	64.70 ± 12.69	69.69 ± 12.89	62.91 ± 12.17	0.002
Male sex, *n* (%)	113 (66.5)	31 (68.9)	82 (65.6)	0.689
Vascular disease risk factors				
Arterial hypertension	107 (62.9)	29 (64.6)	78 (62.4)	0.808
Diabetes mellitus	38 (22.4)	12 (26.7)	26 (20.8)	0.418
Hyperlipidemia	37 (21.8)	10 (22.2)	27 (21.6)	0.931
Smoking	62 (36.5)	10 (22.2)	52 (41.6)	0.021
History of coronary artery disease	34 (20.0)	10 (22.2)	24 (19.2)	0.664
Arterial fibrillation	87 (51.2)	26 (57.8)	61 (48.8)	0.302
History of ischemic stroke	36 (21.2)	6 (13.3)	30 (24.0)	0.133
Pre‐stroke mRS^a^	0 (0–3)	0 (0–3)	0 (0–2)	0.718
Admission blood pressure, mmHg				
Systolic blood pressure	141 ± 23	143.18 ± 26.34	140.31 ± 21.91	0.478
Diastolic blood pressure	83 ± 18	80.78 ± 16.28	84.80 ± 18.07	0.192
Body Mass Index	24.7 ± 3.0	24.54 + ±3.33	24.77 ± 2.86	0.706
Laboratory parameters at admission				
Triglycerides, mmol/L	1.26 ± 0.73	1.20 ± 0.42	1.28 ± 0.80	0.636
Total cholesterol, mmol/L	3.92 ± 1.02	3.87 ± 0.99	3.93 ± 1.03	0.808
LDL cholesterol, mmol/L	2.48 ± 0.91	2.56 ± 1.01	2.46 ± 0.88	0.638
HDL cholesterol, mmol/L	1.22 ± 0.36	1.22 ± 0.39	1.23 ± 0.35	0.954
Plasma glucose, mmol/L	7.48 ± 2.72	7.95 ± 2.77	7.32 ± 2.69	0.192
Platelet count, ×10^9^/L	198.36 ± 59.35	189.84 ± 64.89	201.44 ± 57.17	0.263
Fibrinogen, g/L	3.18 ± 1.05	3.30 ± 1.07	3.13 ± 1.04	0.365
Medication pre‐stroke				
Antiplatelet drugs	43 (25.3)	13 (28.9)	30 (24.0)	0.518
Statins	41 (24.1)	14 (31.1)	27 (21.6)	0.201
Cause of stroke				
Large‐artery occlusion	67 (39.4)	15 (33.3)	52 (41.6)	0.331
Cardioembolic occlusion	89 (52.4)	27 (60.0)	62 (49.6)	0.231
Undetermined or other	14 (8.2)	3 (6.7)	11 (8.8)	0.655
Admission NIHSS score	14 (11–17)	14.5 (11–19)	13.0 (10–17)	0.069
Treated with IVT	57 (33.5)	13 (28.9)	44 (35.2)	0.495
ASPECTS	9 (8–10)	9 (8–10)	9 (8–10)	0.491
Time from onset to Recanalization	400 (311–490)	358 (301–442)	409 (322–518)	0.067

*Note*: Data are mean ± standard deviation, median (interquartile range), or frequency (percent).

Abbreviations: ASPECTS, Alberta stroke program early CT score; HDL, high density lipoprotein; IVT, intravenous thrombolysis; LDL, low density lipoprotein; MCA, middle cerebral artery; NHISS, NIH stroke scale; PI, pulsatility index.

^a^ Data are median (range).

### 3.2 Correlation between the MCA‐PI and functional outcomes

**FIGURE 1:**
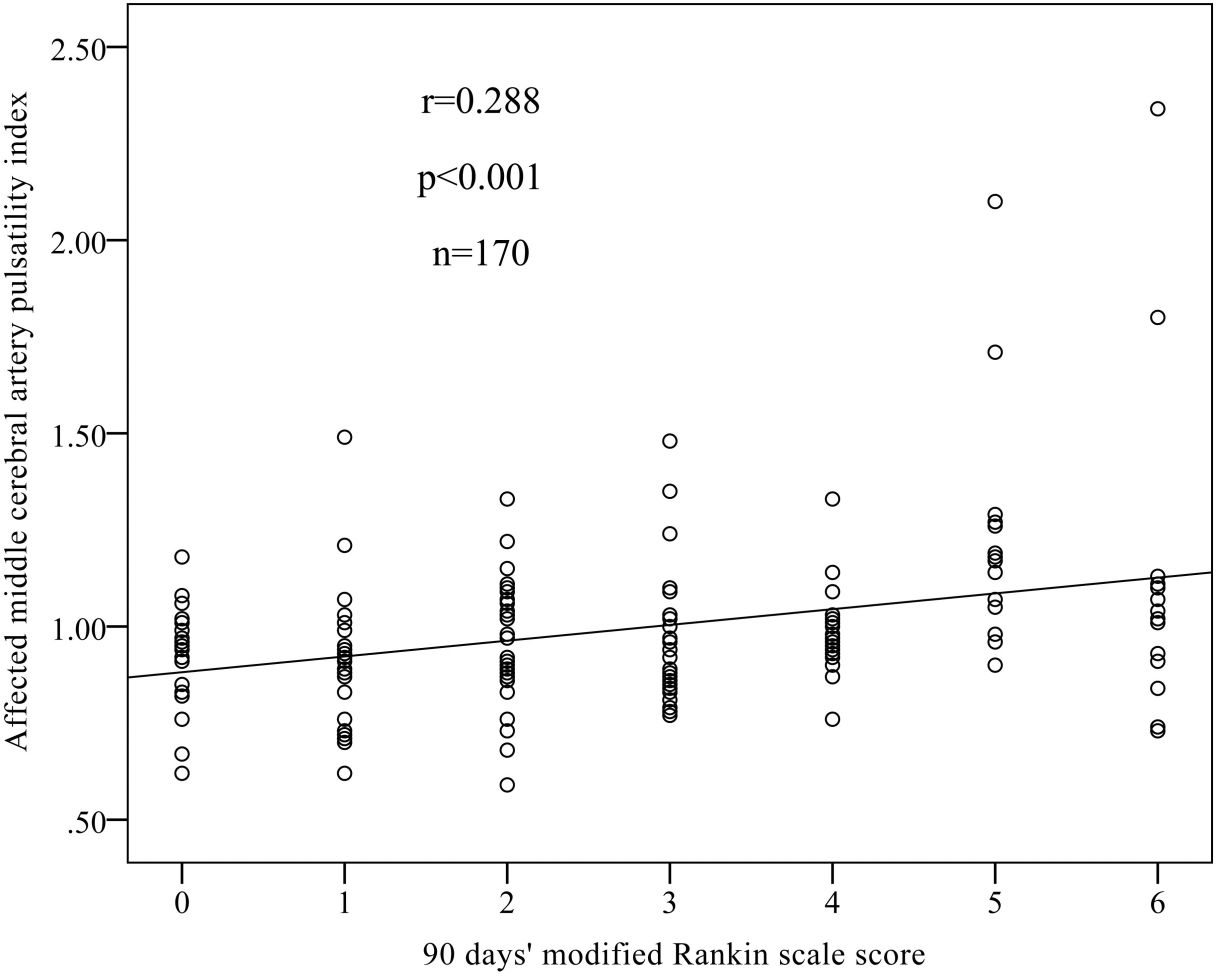
Correlation of pulsatility index and functional outcomes. Correlation of the affected middle cerebral artery (MCA) pulsatility index (PI) with 90‐day functional outcomes assessed by modified Rankin scale (mRS). A total of 170 participants were included, and *r* = 0.288 with *p* < 0.001

The functional outcomes were assessed using the mRS score at 3 months after EVT. The median mRS score was 2.0 (IQR, 1.0–4.0). A total of 51 participants (30.0%) achieved excellent outcomes (mRS score of 0–1); 88 participants (51.8%) achieved functional independence (mRS score of 0–2); and 17 participants (10.0%) died. The MCA‐PI on the affected side was positively correlated with the worsening functional outcomes (assessed by mRS scores) at 3 months after EVT (*r* = 0.204, *p* = 0.008) (Figure 1).

### 3.3 Clinical outcomes

The functional outcomes were also compared between the groups dichotomized according to quartiles (Table 2). The median mRS scores were 4 (IQR, 2–5) in the participants in the highest quartile and 2 (IQR, 1–4) in those in the lower three quartiles. These scores showed significant differences (adjusted *p* = 0.008). The participants in the highest quartile were more likely to experience poor functional outcomes than those in the lower three quartiles (mRS score of 5–6: 42.2% vs. 10.4%; OR, 6.30; 95 CI, 2.76–14.36; *p* < 0.001; mRS score of 4–6: 51.1% vs. 25.6%; OR, 3.04; 95% CI, 1.50–6.18; *p* = 0.002). After adjustments for confounding factors, the highest quartile was significantly associated with a higher ratio of mRS score of 5–6 (adjusted OR, 13.33; 95% CI, 2.65–67.17; adjusted *p* = 0.002) (Table 2).

**TABLE 2:** Clinical outcomes of participants according to pulsatility index quartiles

Patients	Total	MCA‐PI		Unadjusted OR (95% CI)	Unadjusted *p*	Adjusted OR (95% CI)	Adjusted *p*
		Highest quartile	Lower 3 quartiles				
	170	45	125				
Any ICH	48 (28.2)	18 (40.0)	30 (24.0)	2.11 (1.02–4.36)	0.041	1.83 (0.72–4.64)^a^	0.206^a^
Symptomatic ICH	15 (8.8)	6 (13.3)	9 (7.2)	1.98 (0.66–2.93)	0.214	1.26 (0.32–2.05)^a^	0.743^a^
mRS	2 (1–4)	4 (2–5)	2 (1–4)	‐‐	<0.001	‐‐	0.008
6	17 (10.0)	8 (17.8)	9 (7.2)	2.79 (1.00–7.74)	0.053	2.55 (0.32–20.11)^b^	0.376^b^
5–6	32 (18.8)	19 (42.2)	13 (10.4)	6.30 (2.76–14.36)	<0.001	13.33 (2.65–67.17)^b^	0.002^b^
4–6	55 (32.4)	23 (51.1)	32 (25.6)	3.04 (1.50–6.18)	0.002	3.10 (0.97–9.96)^b^	0.057^b^

*Note*: Data are frequency (percent) or median (interquartile range).

Abbreviations: CI, confidence interval; ICH, intracerebral hemorrhage; mRS, modified Rankin scale; OR, odds ratio; PI, pulsatility index.

^a^ Adjusted for age, NIH stroke scale, intravenous thrombolysis, Alberta Stroke Program Early CT Score, onset to recanalization time, hypertension, diabetes mellites, and antiplatelet treatment pre‐stroke.

^b^ Adjusted for age, pre‐stroke modified Rankin scale, NIH stroke scale, intravenous thrombolysis, Alberta Stroke Program Early CT Score, onset to recanalization time, and symptomatic intracerebral hemorrhage.

In total, 48 participants (28.2%) experienced ICH, and 15 (8.8%) experienced symptomatic ICH; 18 participants (40.0%) in the highest quartile and 30 participants (24.0%) in the lower three quartiles experienced ICH, which was significantly different (OR, 2.11; 95% CI, 1.02–4.36; *p* = 0.041). However, after adjustments for confounding factors, the difference became insignificant (adjusted OR, 1.83; 95% CI, 0.72–4.64; adjusted *p* = 0.206). Six participants (13.3%) in the highest quartile experienced symptomatic ICH compared with nine participants (7.2%) in the lower three quartiles, which was insignificantly different (adjusted OR, 1.26; 95% CI, 0.32–2.05; adjusted *p* = 0.743).

### 3.4 ROC curve

**FIGURE 2:**
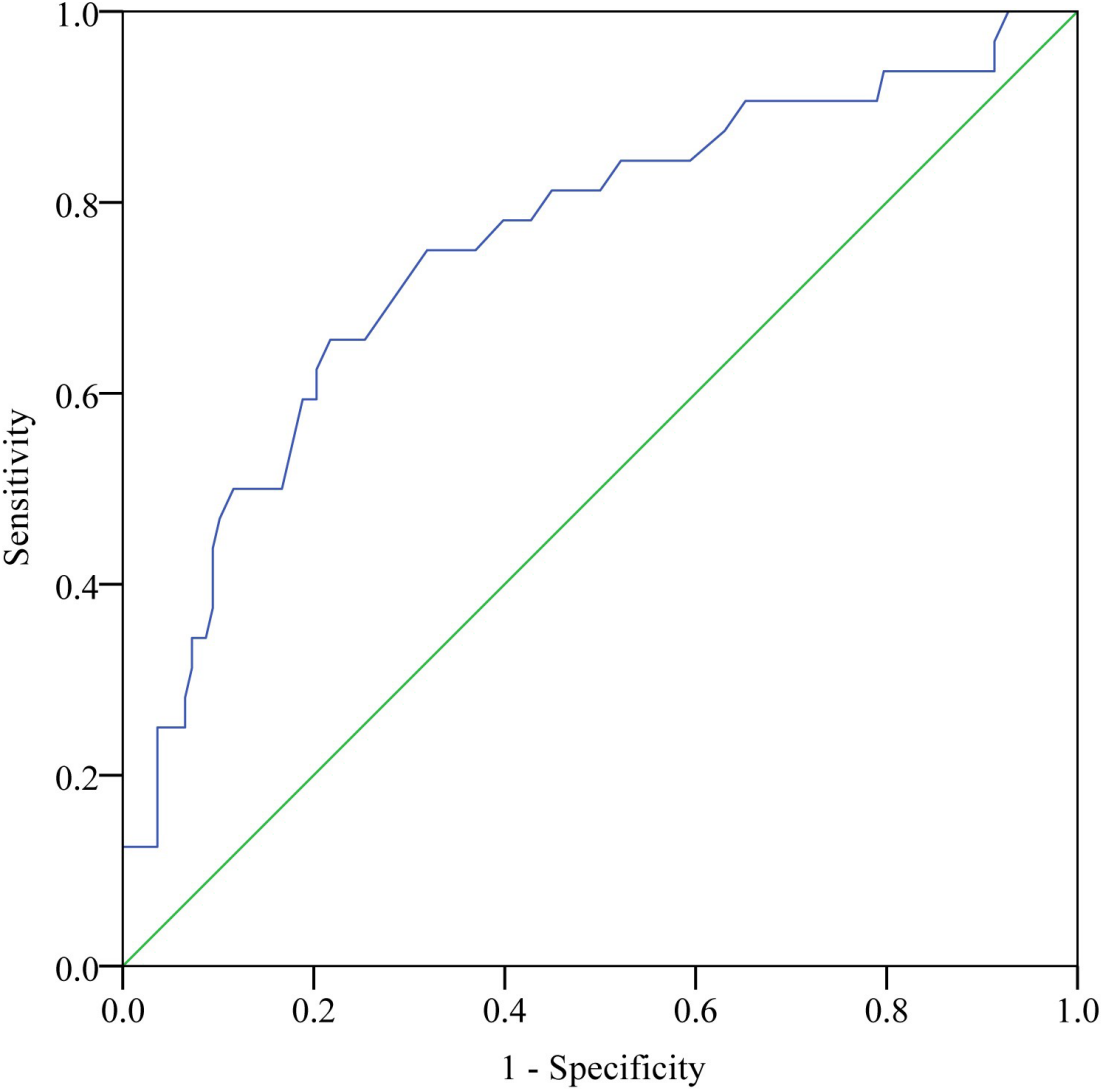
Receiver operating characteristic curve for extremely poor functional outcomes 3 months after thrombectomy demonstrating area under cure was 0.755 (95% confidence interval, 0.655–0.854) with a *p* value < 0.001. The optimal cutoff point of affected middle cerebral artery pulsatility index for predicting poor outcome was 1.04, with sensitivity of 0.656 and specificity of 0.783

The highest quartile was significantly associated with a higher ratio of mRS score of 5–6, which indicated an extremely poor functional outcome. ROC curve and Youden index analyses were performed. The optimal cutoff value of the MCA‐PI on the affected side for predicting extremely poor functional outcomes was projected to be 1.04, which yielded a sensitivity of 65.6% and a specificity of 78.3%, with an area under the curve of 0.755 (95% CI, 0.655–0.854; *p* < 0.001) (Figure 2).

### 3.5 Risk factors of MCA‐PI on the affected side

Linear regression analysis was performed to adjust for the possible confounders of the MCA‐PI on the affected side (Table 3). Apart from male sex, smoking, baseline plasma fibrinogen level, pretreatment with statins, and stroke type according to the TOAST criteria, the MCA‐PI on the affected side was found to be independent of age, hypertension, diabetes mellitus, hyperlipidemia, baseline NIHSS score, treatment with intravenous thrombolysis, symptomatic ICH, and baseline laboratory parameters, such as plasma glucose level and platelet count.

**TABLE 3:** Multifactorial analysis of covariance for pulsatility index

Factors	Effect size (F)	*p* Value
Age	0.144	0.092
Male sex	0.236	0.013
Vascular disease risk factors		
Arterial hypertension	0.037	0.676
Diabetes mellitus	0.036	0.677
Hyperlipidemia	−0.019	0.832
Smoking	−0.224	0.013
Laboratory parameters at admission		
Plasma glucose	0.152	0.072
Platelet count	−0.038	0.656
Fibrinogen	0.186	0.033
Medication pre‐stroke		
Antiplatelet drugs	−0.097	0.298
Statins	0.247	0.016
Admission NIHSS score	0.154	0.067
Treated with IVT	0.056	0.494
Symptomatic ICH	0.037	0.638
TOAST type of ischemic stroke	0.188	0.030

Abbreviations: ICH, intracerebral hemorrhage;IVT, intravenous thrombolysis; NIHSS, NIH stroke scale; TOAST, trial of ORG 10172 in acute stroke treatment.

## 4 DISCUSSION

In this study, we found that in patients with AIS secondary to MCA occlusion in the M1 segment who were treated with EVT and achieved excellent recanalization (TICI grade 3), an elevated MCA‐PI on the affected side was associated with the mRS score at 3 months after EVT. It was predictive of extremely poor functional outcomes based on a mRS score of ≥5, and its optimal cutoff value was 1.04, with a sensitivity of 65.6% and a specificity of 78.3%.

Consistent with previous studies that found that an elevated PI was associated with worse functional outcomes after reperfusion therapies,^14^, ^15^ this study found that an elevated PI was associated with the mRS scores and predicted extremely poor functional outcomes. However, in a previous study, participants with TICI grade 2b/3 were recruited; the evaluation of microvascular resistance therein may not have been reliable because TICI grade 2b refers to the presence of ≤1/3 of the residual perfusion deficits in the vascular territory of the initially occluded vessel and it is not fully reperfused.^19^ Thus, the evaluation of patients with TICI grade 2b inevitably leads to an elevated PI. In our study, complete recanalization was considered a key inclusion criterion to reliably assess the association between the PI and outcomes.

In contrast to a study that reported the results of TCD performed within 72‐hour post‐EVT among patients with AIS who underwent thrombectomy,^14^ this study evaluated the results of patients who underwent TCD within 24 hours after thrombectomy. The infarct volume often reaches its maximum days after stroke onset, followed by maximizing of peri‐infarction edema, both of which may lead to increased intracranial pressure and consequently abnormal hemodynamics or an elevated PI.^20^, ^21^, ^22^, ^23^, ^24^ Therefore, an elevated PI assessed for >24 hours after EVT may be attributed to the increased intracranial pressure secondary to large infarct volume, cerebral edema, or hemorrhagic transformation, which seems unreliable for evaluating microvascular resistance. Moreover, in our study, the multifactorial analysis of covariance showed that the MCA‐PI was independent of intravenous thrombolysis, stroke severity assessed using the baseline NIHSS score, and symptomatic ICH; this finding may further confirm the reliability of the MCA‐PI in evaluating microvascular resistance early after EVT.

In addition, a previous study has found that an elevated MCA‐PI was associated with less hemorrhagic infarction^14^; conversely, our study found that it was not associated with either any type of ICH or symptomatic ICH after adjusting for other confounders. The main reasons for this discrepancy may be attributed to the different study populations recruited by the two studies, as the inclusion criteria for TICI were grade 2b/3 in the previous study and grade 3 in this study. This may have also led to the differences in the incidence of ICH reported in these two studies (28.2% vs. 39.6%, respectively). In addition, the previous study has only included a small number of patients (*N* = 53), and the results were not adjusted for potential confounders; this may have resulted in potential biases to their results and differences with the results of our study.

Our study showed that an elevated MCA‐PI was positively correlated with the mRS score, and its highest quartile was significantly associated with extremely poor functional outcomes instead of favorable outcomes. This phenomenon may be attributed to the fact that the PI is a measurement of microvascular resistance and a biomarker of the no‐reflow phenomenon after EVT.^11^, ^12^, ^13^, ^14^ In patients in whom complete perfusion was achieved after recanalization of proximal large vessels, microvascular reperfusion is one of the most important determinants of the final infarct volume and functional outcomes.^25^, ^26^, ^27^ The incidence of the no‐reflow phenomenon is supposed to be much higher in patients with an elevated PI than in their counterparts; furthermore, the higher the PI, the more severe the microvascular resistance. Therefore, an elevated MCA‐PI in the culprit territory after EVT is a predictor of worse functional outcomes.

This study had several limitations. First, this study showed that an elevated MCA‐PI on the affected side was associated with worse outcomes, which was supposed to be related to microvascular no‐reperfusion, but there are no imaging results that support this speculation, despite previous studies having established the PI as an index of cerebrovascular resistance. Further imaging studies that confirm these findings are warranted. Second, as a large infarct volume may alter the PI through increased intracranial pressure, the infarct volume was not included as a covariate when analyzing the confounders of the PI and other results in the study. However, the baseline NIHSS score and ASPECTS, which are markers of stroke severity equally as important as the infarct volume, were included, and they were found to be independent of the PI. Third, the retrospective nature and the small sample size of this study may have caused biases; thus, larger prospective studies are required to confirm our results.

## 5 CONCLUSIONS

An elevated MCA‐PI on the affected side is associated with poor functional outcomes in patients with AIS caused by MCA occlusion who have undergone EVT. Further large‐scale imaging studies are needed to confirm the association between the roles of an elevated MCA‐PI and no‐reflow phenomenon after EVT and substantiate the MCA‐PI as a marker in patients with AIS who have undergone EVT.

## AUTHOR CONTRIBUTIONS

Wenbo Zhao contributed to the study concept and design, statistical analysis, interpretation of data, and drafting and revising the manuscript. Ran Liu contributed to the study concept and design, acquisition, and interpretation of data, drafting and revising the manuscript. Wantong Yu, Longfei Wu, Chuanjie Wu, Chuanhui Li, Sijie Li, and Jian Chen contributed to the acquisition and interpretation of data and revising the manuscript. Haiqing Song contributed to the interpretation of data and revising the manuscript. Yang Hua contributed the study design and supervision, analysis, interpretation of data, and revising the manuscript. Qingfeng Ma contributed to the study concept and design, analysis, interpretation of data, and revising the manuscript. Xunming Ji contributed to the study concept, design, and supervision, statistical analysis, interpretation of data, and drafting and revising the manuscript.

## CONFLICT OF INTEREST

None.

## Data Availability

The data, analytic methods, and study materials of this study are available from the corresponding author on reasonable request.
